# Giant cystic meconium peritonitis presenting in a neonate with classic radiographic eggshell calcifications and treated with an elective surgical approach: a case report

**DOI:** 10.1186/1752-1947-6-229

**Published:** 2012-08-02

**Authors:** Erik R Barthel, Allison L Speer, Daniel E Levin, Bindi J Naik-Mathuria, Tracy C Grikscheit

**Affiliations:** 1 Children’s Hospital Los Angeles Division of Pediatric Surgery, 4650 Sunset Boulevard Mailstop 35, Los Angeles, CA 90027, USA; 2 Pediatric Surgery, Texas Children’s Clinical Care Center, 6701 Fannin Street, 8th Floor, Houston, TX 77030, USA

## Abstract

**Introduction:**

Giant cystic meconium peritonitis is relatively rare. Patients often present with nonspecific physical findings such as distension and emesis. Plain abdominal films remain invaluable for identifying the characteristic calcifications seen with a meconium pseudocyst, and large eggshell calcifications are pathognomonic for the giant cystic subtype.

**Case presentation:**

We present classic plain X-ray findings and an intraoperative image of a premature low birth weight two-day-old Hispanic male baby treated for giant cystic meconium peritonitis with a staged procedure involving peritoneal drainage, ostomy creation and closure.

**Conclusion:**

Pediatric surgeons have a range of potential therapeutic approaches for giant cystic meconium peritonitis. A delay of definitive surgical management in the setting of massive abdominal soiling is a safe and acceptable strategy if adequate temporizing drainage is performed in the early perinatal period.

## Introduction

**Figure 1:**
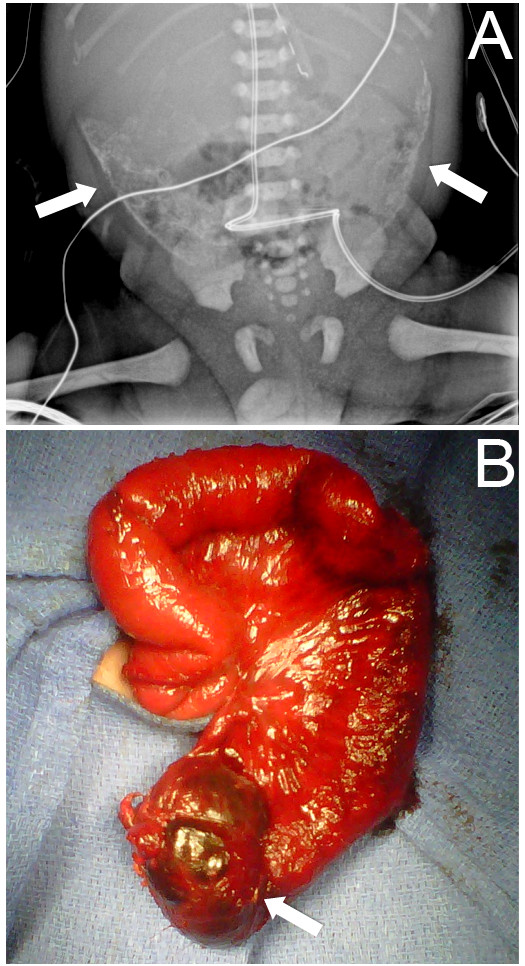
**Initial radiographic image and photograph taken after patient’s second operation.** ( **A**) Plain abdominal radiograph showing bilateral eggshell calcifications consistent with giant cystic meconium peritonitis (GCMP, arrows). ( **B**) Photograph taken at the patient’s second operation, where the peritoneal drain was removed and an ileostomy was brought out. The remnant of the original meconium pseudocyst can be seen at the bottom of the figure (arrow).

Reports of meconium peritonitis appear in the English-language literature beginning in the early 20th century [1,2]. Infants may present with the failure to pass meconium, abdominal distension, emesis and symptoms of peritonitis [3]. Meconium pseudocyst occurs as a result of *in utero* or postnatal intestinal perforation. This perforation is often secondary to ischemia or obstruction and although meconium is sterile, chemical irritation of the peritoneum can ensue [4]. As the body attempts to wall off the source of inflammation, dystrophic calcification occurs, resulting in the classic eggshell calcifications seen on abdominal radiography, demonstrated in Figure 1[5].

## Case presentation

A 2.2 kg Hispanic boy was born vaginally to a 21 year-old primigravida woman at 31 weeks and one day of gestation after prolonged rupture of membranes. He had a prenatal history of dilated bowel loops and hydrops on ultrasonography, as well as fetal anemia requiring an intrauterine blood transfusion 18 days prior to delivery. Our patient was intubated at 11 minutes of life for poor respiratory effort. Analysis of his cord arterial blood gas on transfer to the neonatal intensive care unit revealed a pH of 7.32, partial pressure of CO_2_ 50 torr, and partial pressure of O_2_ 20. His abdomen was markedly distended, exhibited progressive erythema, and radiographs showed dilated bowel loops and significant, well-demarcated bilateral calcifications (Figure 1A, arrows).

Our patient was diagnosed with a giant meconium pseudocyst, a walled-off collection of meconium that can cause neonatal peritonitis, which is distinguished from other forms of meconium peritonitis by the persistence of a communication between the digestive lumen and the peritoneal cavity leading to the entire abdomen becoming filled with meconium [6]. On the second day of life, he was taken to the operating room, placed in the supine position, and an exploratory laparotomy was performed. At surgery, extensive soiling and inflammation precluded the identification of intestinal loops for safe stoma formation. Bilateral Penrose drains were therefore placed as a temporizing measure to allow decompression of the meconium from his abdomen.

Although our patient’s sepsis improved with source control, he remained obstructed with a high nasogastric output. He also had persistent impairment of his pulmonary function from the obstruction and abdominal distension. Therefore, he was taken for a second operation one month later. In this procedure, an extensive lysis of adhesions was performed, revealing a meconium cyst remnant (Figure 1B). His distal microcolon was also visualized, and an ileostomy was brought out at this time. Feeds were started on postoperative day 11 and were slowly advanced over the next four weeks to goal. Our patient was discharged and continued to gain weight appropriately at home. A screening chloride sweat test for cystic fibrosis was performed at four months of life and was negative. He was brought back in for elective ileostomy closure at seven months of life, at which time he was thriving on oral feeds and was weaned off parenteral nutrition. We also note that, to date, he has had no episodes of adhesive bowel obstruction or any evidence of blind loop syndrome.

## Discussion

Broadly, the meconium syndromes constitute a spectrum of diseases, all of which can present with neonatal bowel obstruction [7]. These may include meconium ileus, which is often associated with cystic fibrosis and can be amenable to nonoperative management with saline, contrast or *N*-acetylcysteine enema administration, but requires surgical intervention if such measures fail [8]. In fact, it has recently been reported that contrast enema therapy for a simple meconium ileus has become less successful over time, requiring more frequent operative intervention. These authors attribute this effect to the changing practice pattern of radiologists and differing properties of various enema agents that have been employed [9]. Meconium plug syndrome is a related but distinct entity characterized by colonic obstruction by inspissated meconium. It is usually benign, resolves with contrast enema administration, and is not usually associated with cystic fibrosis [10]. In turn, a distinction must be made between this condition and small left colon syndrome, which is associated with maternal diabetes, but also typically resolves with rectal contrast administration [11].

The defining characteristic of meconium pseudocyst and meconium peritonitis, which distinguishes these conditions from meconium ileus or meconium plug syndrome, is intrauterine perforation with extralumenal leakage of enteric material into the peritoneal cavity [12]. Outcomes for neonatal meconium peritonitis have improved over time, with relatively high mortality rates reported from the 1950s improving to a survival rate of about 90% with modern advances in pediatric surgery and perinatology [13]. Surgery remains the definitive treatment, but there is a lack of standardization in the literature. Multiple drainage procedures, stoma creation and final ostomy closure are still common [14]. Of note, a recent retrospective series of 38 patients demonstrated improved survival with primary anastomosis, except for low birth weight babies [9]. Our patient had extensive soilage and adhesions that required a temporary drainage procedure, and definitive stoma creation was delayed for one month. A similar strategy has been reported previously [15]. These authors presented two cases in which they placed external drains under ultrasound guidance, and then waited 16 and 20 days, respectively, before proceeding to laparotomy. They elected to perform a primary ileal anastomosis at surgery in both patients. However, we chose to bring out a stoma at delayed laparotomy given their patient’s prolonged neonatal intensive care unity stay and sepsis course. While our choice of stoma creation, rather than primary anastomosis, is consistent with the recommendations of Copeland *et al*. [9] given the birth weight of 2.2 kg, we stress that our decision to start with a temporary drainage procedure prior to definitive surgery is more consistent with the initial management by Ellis *et al*. [11].

## Conclusion

A classic appearance of large calcifications on plain abdominal films in the setting of a distended and distressed neonate should place giant cystic meconium peritonitis high upon the differential diagnosis. Initial peritoneal drainage with a delay in definitive surgical management is a reasonable approach in the setting of massive meconium soiling of the abdominal cavity that prohibits the safe identification of bowel loops for stoma creation. At reoperation, bringing out a stoma is a safe option, especially for low birth weight infants.

## Consent

Written informed consent was obtained from the patient’s next-of-kin for publication of this case report and any accompanying images. A copy of the written consent is available for review by the Editor-in-Chief of this journal.

## Competing interests

The authors declare that they have no competing interests.

## Authors’ contributions

ERB, ALS and DEL performed the literature search and wrote the manuscript. BJN-M and TG performed the surgeries, provided intraoperative images and critically reviewed the manuscript prior to submission. All authors have read and approved the final manuscript.
